# Downregulation of *DmMANF* in Glial Cells Results in Neurodegeneration and Affects Sleep and Lifespan in *Drosophila melanogaster*

**DOI:** 10.3389/fnins.2017.00610

**Published:** 2017-11-02

**Authors:** Lucyna Walkowicz, Ewelina Kijak, Wojciech Krzeptowski, Jolanta Górska-Andrzejak, Vassilis Stratoulias, Olga Woznicka, Elzbieta Chwastek, Tapio I. Heino, Elzbieta M. Pyza

**Affiliations:** ^1^Department of Cell Biology and Imaging, Institute of Zoology and Biomedical Research, Jagiellonian University, Krakow, Poland; ^2^Department of Biosciences, University of Helsinki, Helsinki, Finland

**Keywords:** MANF, CDNF, neurotrophic factor, visual system, neuroprotection, neurodegeneration

## Abstract

In *Drosophila melanogaster*, mesencephalic astrocyte-derived neurotrophic factor (DmMANF) is an evolutionarily conserved ortholog of mammalian MANF and cerebral dopamine neurotrophic factor (CDNF), which have been shown to promote the survival of dopaminergic neurons in the brain. We observed especially high levels of DmMANF in the visual system of *Drosophila*, particularly in the first optic neuropil (lamina). In the lamina, DmMANF was found in glial cells (surface and epithelial glia), photoreceptors and interneurons. Interestingly, silencing of *DmMANF* in all neurons or specifically in photoreceptors or L2 interneurons had no impact on the structure of the visual system. However, downregulation of *DmMANF* in glial cells induced degeneration of the lamina. Remarkably, this degeneration in the form of holes and/or tightly packed membranes was observed only in the lamina epithelial glial cells. Those membranes seem to originate from the endoplasmic reticulum, which forms autophagosome membranes. Moreover, capitate projections, the epithelial glia invaginations into photoreceptor terminals that are involved in recycling of the photoreceptor neurotransmitter histamine, were less numerous after *DmMANF* silencing either in neurons or glial cells. The distribution of the alpha subunit of Na+/K+-ATPase protein in the lamina cell membranes was also changed. At the behavioral level, silencing of *DmMANF* either in neurons or glial cells affected the daily activity/sleep pattern, and flies showed less activity during the day but higher activity during the night than did controls. In the case of silencing in glia, the lifespan of flies was also shortened. The obtained results showed that DmMANF regulates many functions in the brain, particularly those dependent on glial cells.

## Introduction

Neurotrophic factors (NTFs) play a crucial role in development of the central nervous system as well as in shaping many aspects of nerve cell metabolism and physiology in the mature brain of vertebrates. They regulate the number, differentiation and plasticity of neurons (Huang and Reichardt, 2001). Moreover, NTFs protect neurons against apoptosis and support the survival, proliferation, and maturation of certain populations of neurons (Huang and Reichardt, 2001; Lindholm and Saarma, 2010; Ceni et al., 2014; Tang et al., 2017). Two novel, evolutionarily conserved NTFs, mesencephalic astrocyte-derived neurotrophic factor (MANF) (Petrova et al., 2003) and cerebral dopamine neurotrophic factor (CDNF, paralog of MANF) (Lindholm et al., 2007), have been reported to support the survival of dopaminergic neurons. Because of their neuroprotective properties and their expression in the striatum receiving dopaminergic projections (Lindholm et al., 2007, 2008; Voutilainen et al., 2009), MANF and CDNF have already been tested as neuroprotective factors in animal models of Parkinson's disease (Lindholm et al., 2007; Voutilainen et al., 2009; Airavaara et al., 2012). MANF, CDNF, and other NTF-based drugs, which can restore degenerating cells in the brain, appear to be promising alternative therapies. They can reduce symptoms or even restore neuronal functions in neurodegenerative diseases (reviewed in Domanskyi et al., 2015; Voutilainen et al., 2015).

In *Drosophila melanogaster*, Palgi et al. (2009) described a single protein homologous to CDNF/MANF. Because the primary structure of the protein shared approximately 50% identity with that of human MANF, it was called DmMANF (*Drosophila melanogaster* MANF). DmMANF is required for the maturation of the embryonic nervous system and the maintenance of dopaminergic neurons. Maternal and zygotic *DmMANF* null mutants are characterized by extremely low levels of dopamine and diminished dopaminergic neurites (Palgi et al., 2009).

Although the molecular mechanisms of DmMANF action are still mostly unknown, its role in the unfolded protein response (UPR) in endoplasmic reticulum (ER) stress has been reported (Palgi et al., 2012; Lindström et al., 2016). UPR contributes to the impairment of ER through downregulation of protein synthesis or degradation of misfolded proteins (Ryoo, 2015). In *DmMANF* mutants the expression of genes related to stress, defense, immune responses, proteolysis, and cell death was upregulated, and more than 30% of all examined genes related to ER and UPR showed altered mRNA levels (Palgi et al., 2012). Recently, it has also been shown that MANF has a conserved immune modulatory function, in both *Drosophila* and mouse, promoting tissue repair and regeneration in the retina (Neves et al., 2016).

*DmMANF* is expressed not only in neuronal but also in various non-neuronal tissues in all developmental stages of *D. melanogaster* (Palgi et al., 2009, 2012; Stratoulias and Heino, 2015a; Lindström et al., 2017). Interestingly, DmMANF was found only in glial cells in the embryonic nervous system (Palgi et al., 2009), whereas in the brain of adult *D. melanogaster* its expression is more widely distributed, in both glial cells and neurons (Stratoulias and Heino, 2015a). In contrast to glia, where DmMANF is in somata and processes, in neurons it has been detected only in cell bodies, including the somata of seven clusters of dopaminergic neurons (Stratoulias and Heino, 2015a). The presence of DmMANF in both neurons and glia of the adult nervous system suggests that this protein plays a key role in the nervous system, possibly in neuron–glia interactions.

**Figure 1:**
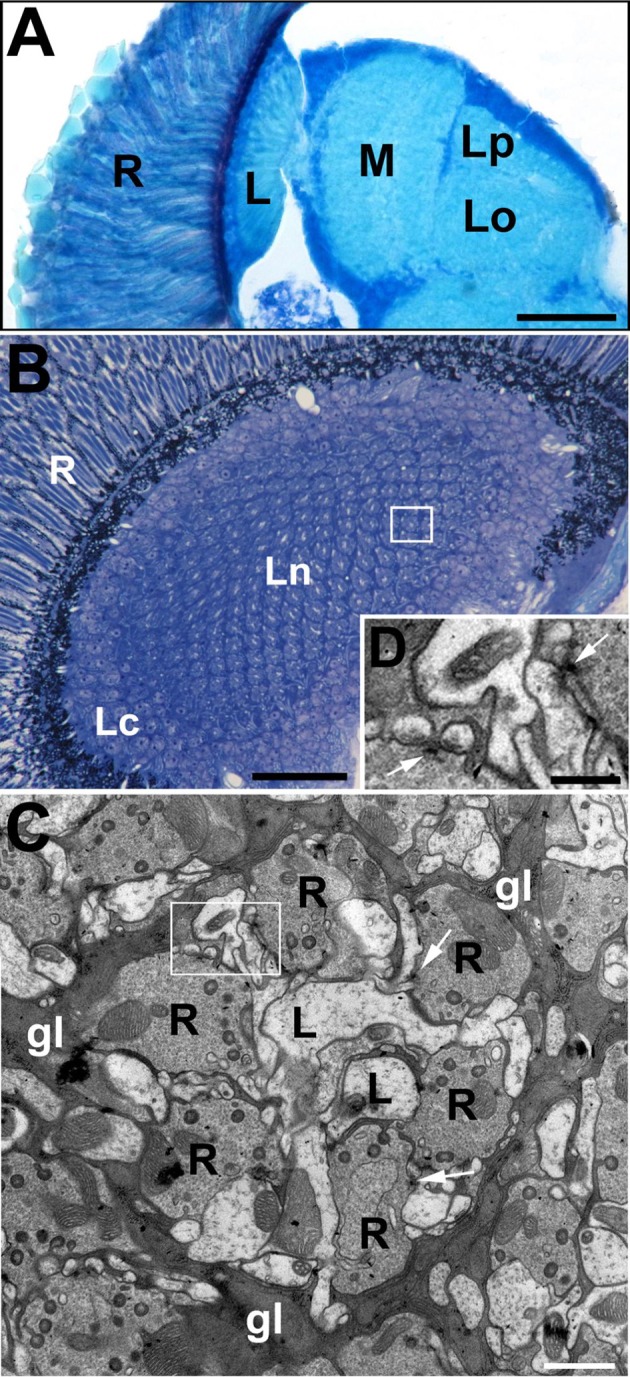
The visual system of the fruit fly, *Drosophila melanogaster*. **(A)** The horizontal section of the compound eye retina (R) and neuropils of the underlying optic lobe: lamina (L), medulla (M) and lobula complex (Lo, lobula; Lp, lobula plate). Scale bar: 50 μm. **(B)** Cross section of the lamina, which is composed of an array of synaptic units called cartridges. A single cartridge is visible in rectangle. R, retina; Lc, lamina cortex; Ln, lamina neuropil. Scale bar: 25 μm. **(C)** Electron microscopy (EM) micrograph of a cartridge. Each cartridge comprises the same types of elements including photoreceptor terminals (R), L1 and L2 (L) monopolar cells (always positioned at the cartridge axis). Cartridges are surrounded by epithelial glial cells (gl). Their processes are filled with mitochondria as well as rough and smooth endoplasmic reticulum. The numerous presynaptic elements (T-bars) in the photoreceptor terminals (arrows) contact four postsynaptic partners forming the so-called tetrad synapses. Scale bar: 1 μm. **(D)** Magnification of the region in the frame on **(C)** shows the T-bars (arrows) in two photoreceptor terminals. Scale bar: 500 nm.

In the present study, we examined the pattern of DmMANF expression and its importance in neurons and glial cells of the *Drosophila* brain, especially in the first neuropil of the visual system (Figure 1). Interactions between neurons and glial cells in this neuropil display high plasticity, including circadian plasticity, which we have reported (Górska-Andrzejak et al., 2009, 2013; Górska-Andrzejak, 2013; Woznicka et al., 2015). Additionally, the lamina is a convenient model for studying various processes in the nervous system due to its regular structure formed by cylindrical units called cartridges (Nériec and Desplan, 2016). Each cartridge (Figure 1C) consists of processes of many cells, including the photoreceptor terminals (R1–R6), L1–L5 monopolar cells, amacrine cells, and processes of cells located in the second optic neuropil (medulla) and in the central brain (Meinertzhagen and Sorra, 2001). In addition, each cartridge is surrounded by three epithelial glial cells, which extend processes into nearby cartridges and invaginate into the photoreceptor terminals (Trujillo-Cenóz, 1965; Stark and Carlson, 1986; Prokop and Meinertzhagen, 2006). These invaginations, the so-called capitate projections, are the sites of neurotransmitter recycling and thus may coordinate photoreceptor-glia communication in the lamina (Fabian-Fine et al., 2003; Rahman et al., 2012; Petralia et al., 2015).

We found that DmMANF is widely expressed in the adult visual system of the fruit fly, which suggests that this NTF has an important functional role in this context. We found that DmMANF is more abundant in the lamina epithelial glial cells, in their cell bodies and processes, rather than in other types of glia and neurons. Moreover, the silencing of *DmMANF* expression in all glial cells induced severe degeneration only in the lamina epithelial glia. Although DmMANF is also present in neurons, the silencing of *DmMANF* only in glia decreased the lifespan of flies. Finally, decreased levels of DmMANF in either glia or neurons affected the sleep and locomotor activity of flies.

## Materials and methods

### Fly strains

We used the following strains of *Drosophila melanogaster*: CantonS, *w*^*1118*^*, repo-*Gal4 (*w*^*1118*^; P{GAL4}repo/TM3, Sb^1^)*, elav-*Gal4 (P{GAL4-elav.L}2/CyO), UAS*-mCD8::GFP* (*y*^1^w^*^; P{UAS-mCD8.mGFP.LG}10b), UAS*-mCD8::RFP* (*y*^1^w^*^; P{UAS-mCD8.mRFP.LG}10b), a line with ubiquitously expressed EYFP tagged with endoplasmic reticulum targeting sequence EYFP-ER (w^*^; P{sqh-EYFP-ER}3) (Bloomington *Drosophila* Stock Centre); UAS*-DmMANF*^*RNAi*^ (v12835 from Vienna Drosophila RNAi Centre, VDRC); UAS*-DmMANF* (Palgi et al., 2009) and *21D-*Gal4 (a kind gift from Dr. Thomas Raabe, see details in Górska-Andrzejak et al., 2005), *GMR-*Gal4, *RH1-*Gal4. Flies were reared on a standard yeast-cornmeal-agar medium under a day/night cycle (LD 12:12; 12 h of light and 12 h of darkness) at 25 ± 1°C.

### Immunohistochemistry and confocal microscopy

For immunohistochemistry on cryostat sections, 1-week-old males were decapitated at the beginning of the day (1 h after lights on) and fixed on ice in 4% paraformaldehyde (PFA) in 0.1 M phosphate buffer (PB) for 4 h. Following fixation, heads were washed in phosphate buffered saline (PBS), cryoprotected in 25% sucrose (overnight at 4°C) and mounted in Tissue-Tek medium (Cryomatrix, Thermo Scientific). Cryosections of 20 μm thickness were cut and immunolabeled with different primary antibodies: rabbit anti-DmMANF Ab (1:1000, Palgi et al., 2009), mouse anti-GFP (1:1000, Novus Biologicals) or mouse anti-α subunit of the Na^+^/K^+^-ATPase (1:50, Developmental Studies Hybridoma Bank). Goat anti-rabbit (1:1000, Molecular Probes Invitrogen) and goat anti-mouse (1:1000, Molecular Probes Invitrogen) antibodies conjugated with Alexa488 Fluor or goat anti-mouse (1:400, Jackson ImmunoResearch Laboratories) antibodies Cy3 conjugated were used as the secondary antibodies. Nuclei were stained with DAPI (1:1000, Molecular Probes). Following immunolabeling, brain sections were washed and mounted in Vectashield medium (Vector Laboratories). For whole brains immunolabeling, heads of 1-week-old males were fixed in 4% PFA in PBS with 0.1% Triton X 100 (0.1% PBS-Tx) for 3 h at room temperature. Next, they were washed two times in PBS for 15 min and once in PBS with 0.5% Triton X 100 (0.5% PBS-Tx) for 15 min. Afterwards, brains were dissected, blocked overnight in 5% Normal Goat Serum in 0.5% PBS-Tx and incubated overnight with rabbit anti-DmMANF (1:1000) primary antibodies at 4°C. The brains were then washed three times in 0.5% PBS-Tx for 15 min and incubated with the secondary antibody, goat anti-rabbit (1:400, Jackson ImmunoResearch Laboratories) conjugated with Cy3, for 3 h at room temperature. In the next step, brains were washed again three times in 0.5% PBS-Tx for 15 min and once in 0.01 M PBS for 15 min, and finally they were mounted in Vectashield medium.

Images were taken using a Zeiss LSM 510 META confocal microscope.

### Western blotting

Adult males (7–10 days old) were frozen in liquid nitrogen (30 flies of each genotype) at the beginning of the day (1 h after lights on). Heads were cut off on ice and manually crushed in liquid nitrogen using a handheld homogenizer (LLG). Then, they were homogenized using an ultrasonic homogenizer (Dr. Hielscher) in 2× Laemmli Buffer with protease inhibitors (Boehringer). Samples were gently shaken, frozen at −20°C, thawed and centrifuged at 13,200 rpm for 1 h at 4°C. The supernatant was denatured at 80°C for 5 min. The protein concentration was measured using a Quant-iT Protein Assay Kit and a Qubit fluorometer (Invitrogen). Electrophoresis was performed using a NuPAGE SDS-PAGE Gel System. A 4–12% Bis-Tris gel (Invitrogen) was loaded with 10 μg of protein. The proteins were blotted by electrotransfer onto a PVDF membrane (Invitrogen) followed by blocking with 5% dry milk in PBS with 0.1% Tween-20 for 1 h at room temperature. Membranes were incubated with rabbit anti-DmMANF (1:5000) and anti-α tubulin (1:20000, Abcam 4074) antibodies as a loading control. For secondary antibody, we used goat anti-rabbit HRP (1:10000, Abcam 6721). The ECL Western blotting detection kit (Advansta) was applied for immunodetection. Densitometric quantification was performed using ImageJ software. The Western blot analysis was repeated in four independent experiments.

### Transmission electron microscopy (TEM)

The heads of 1-week-old males were dissected and fixed in cacodyl-buffered PFA (2.5%) and glutaraldehyde (2%) primary fixative for 1 h. They were post-fixed in OsO_4_ (2%) in veronal acetate buffer for 1 h. Subsequently, the heads were dehydrated in a series of alcohols and propylene oxide and embedded in Poly/Bed 812 resin (Polysciences). Ultrathin sections (65 nm thick) of the lamina were cut and contrasted with uranyl acetate and lead citrate. Images of the lamina ultrastructure were taken using a Jeol JEM 2100 HT TEM. The number of capitate projections in the photoreceptor terminals of experimental and control flies were counted from TEM micrographs.

### Lifespan analysis

Lifespan was examined for male experimental flies with silenced expression of DmMANF in glia (*w*^*1118*^; UAS*-DmMANF*^*RNAi*^*/*+; P{GAL4}repo/+, referred to as *repo*-Gal4>UAS*-DmMANF*^*RNAi*^ in the text below) and in neurons (P{GAL4-elav.L}2/UAS*-DmMANF*^*RNAi*^, referred to as *elav*-Gal4>UAS*-DmMANF*^*RNAi*^ in the text below), as well as for adequate control groups (*w*^*1118*^; P{GAL4}repo/+ and P{GAL4-elav.L}2/+ referred to as *repo*-Gal4/+ and *elav*-Gal4/+ in the text below, respectively). The lifespan examination of each group was conducted every 2 days, and each experiment was repeated three times. The significance of differences between Kaplan-Meier survival plots for the studied groups of flies was analyzed by log-rank test using STATISTICA (Statsoft) software. The median survival of each group indicates the day at which 50% of flies were dead.

### Locomotor activity analysis

Locomotor activity of the experimental flies with silenced expression of *DmMANF* in glia (*repo*-Gal4>UAS*-DmMANF*^*RNAi*^) or neurons (*elav*-Gal4>UAS*-DmMANF*^*RNAi*^) and of two control groups (*repo*-Gal4/+, *elav*-Gal4/+) was recorded using a Drosophila Activity Monitoring system (DAM2; TriKinetics) at 25°C. One- to three-day-old males of the genotypes mentioned above were placed inside glass tubes containing rearing medium. The tubes were loaded into the activity monitors and placed in an incubator where the recording was conducted (Rosato and Kyriacou, 2006). Flies were maintained under LD 12:12, and their activity as recorded on the second day of the experiment was analyzed. Total activity was counted as the sum of readings per day. Since sleep in *Drosophila* is defined as a period of uninterrupted behavioral immobility lasting more than 5 min (Huber et al., 2004), for sleep analysis we counted the number of 5-min bins per hour of fly immobility during the second day of the experiment.

## Results

### DmMANF is present in neurons and glial cells of the *Drosophila* visual system

**Figure 2:**
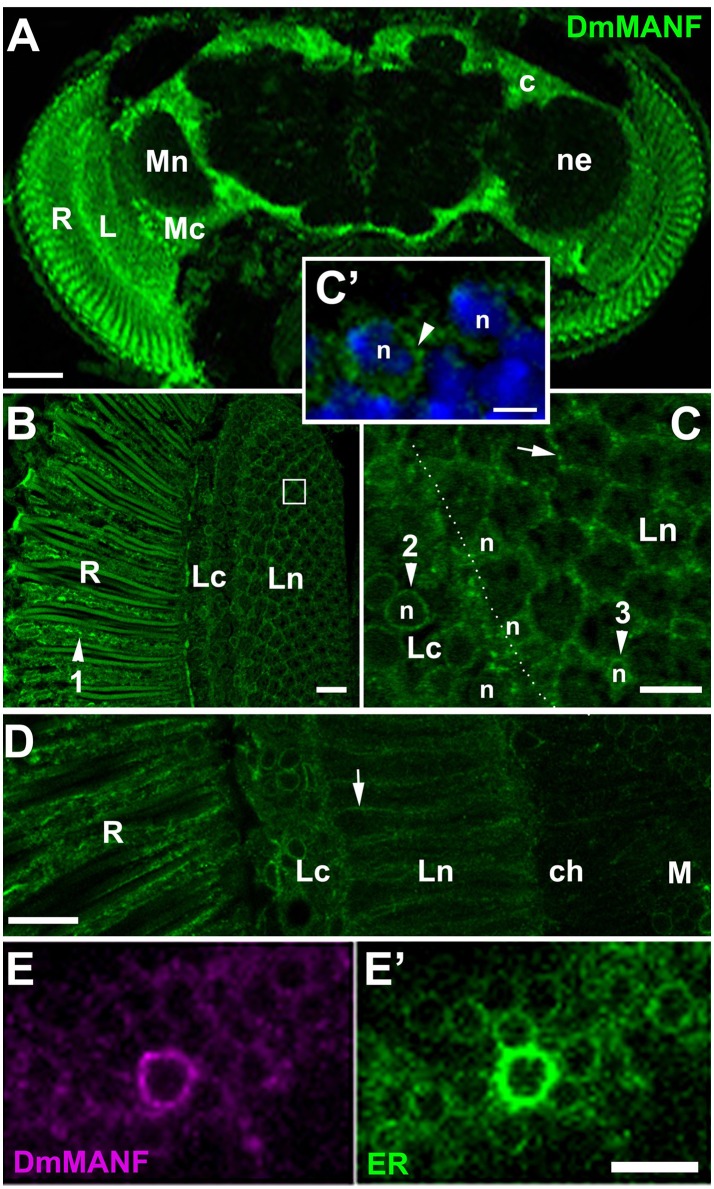
**(A)** DmMANF-specific immunofluorescence in the retina and the brain of *Drosophila melanogaster*. R, retina; L, lamina; Mc, medulla cortex; Mn, medulla neuropil; ne, the region of neuropil; c, the cortex region. Scale bar: 50 μm. **(B)** DmMANF-specific immunofluorescence in the photoreceptor cell bodies (arrowhead 1) of the compound eye retina (R), as well as in different cell types in the lamina cortex (Lc) and neuropil (Ln), in cross section. A single cartridge of the lamina is visible in the white rectangle. Scale bar: 10 μm. **(C)** A fragment of the lamina cortex (Lc) and neuropil (Ln) revealing perinuclear rings (arrowheads 2 and 3, respectively) surrounding cell's nucleus (n). In the distal part of the lamina neuropil (arrowhead 3) reside cell bodies of the epithelial glial cells. A strong punctate pattern of immunofluorescence can also be observed between the cartridges (arrow), in the epithelial glial cell processes. Scale bar: 5 μm. **(C')** Magnification of lamina cortex. Nuclei labeled by DAPI (n) are encircled by DmMANF-specific perinuclear signal (arrowhead). Scale bar: 2 μm. **(D)** The longitudinal section of the lamina neuropil (Ln) reveals the presence of DmMANF-specific immunofluorescence along processes of the epithelial glial cells (arrow). R, retina; ch, chiasm; M, medulla. Scale bar: 10 μm. **(E,E')** The medulla cortex region of EYFP-ER flies revealing the co-localization of DmMANF **(E)** with perinuclear ER **(E')** in numerous cell bodies. The cell with particularly strong DmMANF-specific signal shows the strongest fluorescence of EYFP reporter of ER. Scale bar for **(E,E')**: 5 μm.

Immunolabeling of the brain in strain *w*^*1118*^ revealed the presence of DmMANF in the retina of the compound eye and in the brain (Figure 2A). In the retina, DmMANF was present in the photoreceptor cell bodies (Figure 2B), whereas in the brain it was observed mostly in the neuropil cortical regions (Figure 2A). The signal was particularly strong in case of the first optic neuropil (lamina) underlying the compound eye (Figure 2A), since it was present not only in the lamina cortex but also in the synaptic neuropil. In the lamina cortex, which is composed of densely packed cell bodies of first-order interneurons and glia, DmMANF-positive intracellular structures were found to form characteristic punctate rings encircling the nucleus in numerous cell bodies (Figures 2C,C'). The perinuclear localization of DmMANF, as well as its delicate punctate pattern, suggest its presence in the endoplasmic reticulum. Similar staining was also observed in the perinuclear space of the epithelial glia cell bodies located in the distal part of the lamina neuropil (Figure 2C). More importantly, however, in case of the epithelial glial cells, the DmMANF-specific immunofluorescence was largely located in glial processes that surround the lamina cartridges (Figure 2C) and penetrate the whole depth of the lamina neuropil (Figure 2D). Due to the presence of DmMANF in these glial processes, the staining in the lamina appeared to be particularly strong. In glial processes, similar to cell bodies, the DmMANF-specific immunofluorescence had a subtle punctate pattern (Figures 2C,D), which suggests the intracellular localization of DmMANF at the peripheral ER. The co-localization of DmMANF protein and EYFP-labeled ER was observed in many cells of the fly's brain, including three neuropils of the visual system (Figures 2E,E').

**Figure 3:**
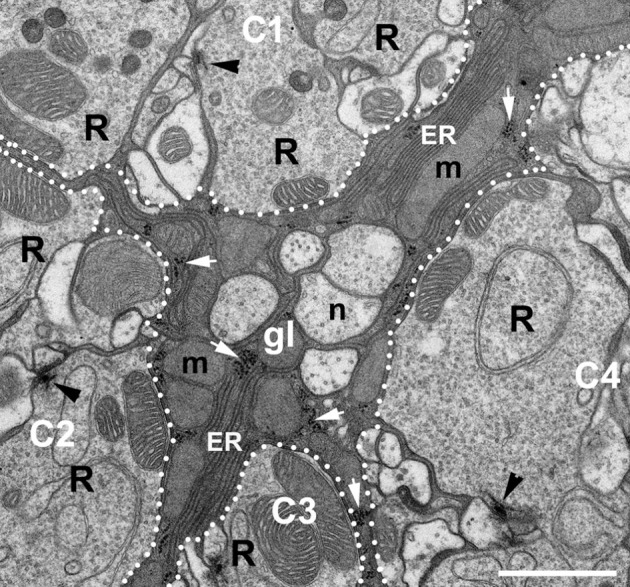
EM micrograph showing the ultrastructure of the epithelial glial cells (gl) placed between four cartridges (C1–C4) of the lamina. Fragments of the cartridges are distinguished from glia by dotted lines. Arrowheads point to the presynaptic elements of tetrad synapses, the so-called T-bars in the photoreceptor terminals (R). The glial processes can be easily distinguished from neuronal elements of cartridges and neuronal processes (n) lying between cartridges by dark cytoplasm. They are filled with mitochondria (m), piles of membranes of endoplasmic reticulum (ER) and ribosomes (white arrows). Scale bar: 1 μm.

TEM micrographs of the lamina ultrastructure showed that ER forms an elaborate network of sheets situated between numerous mitochondria that are densely packed in glial processes enveloping cartridges (Figure 3). Importantly, such complex ER could not be observed in other processes of the cartridge (Figure 3).

**Figure 4:**
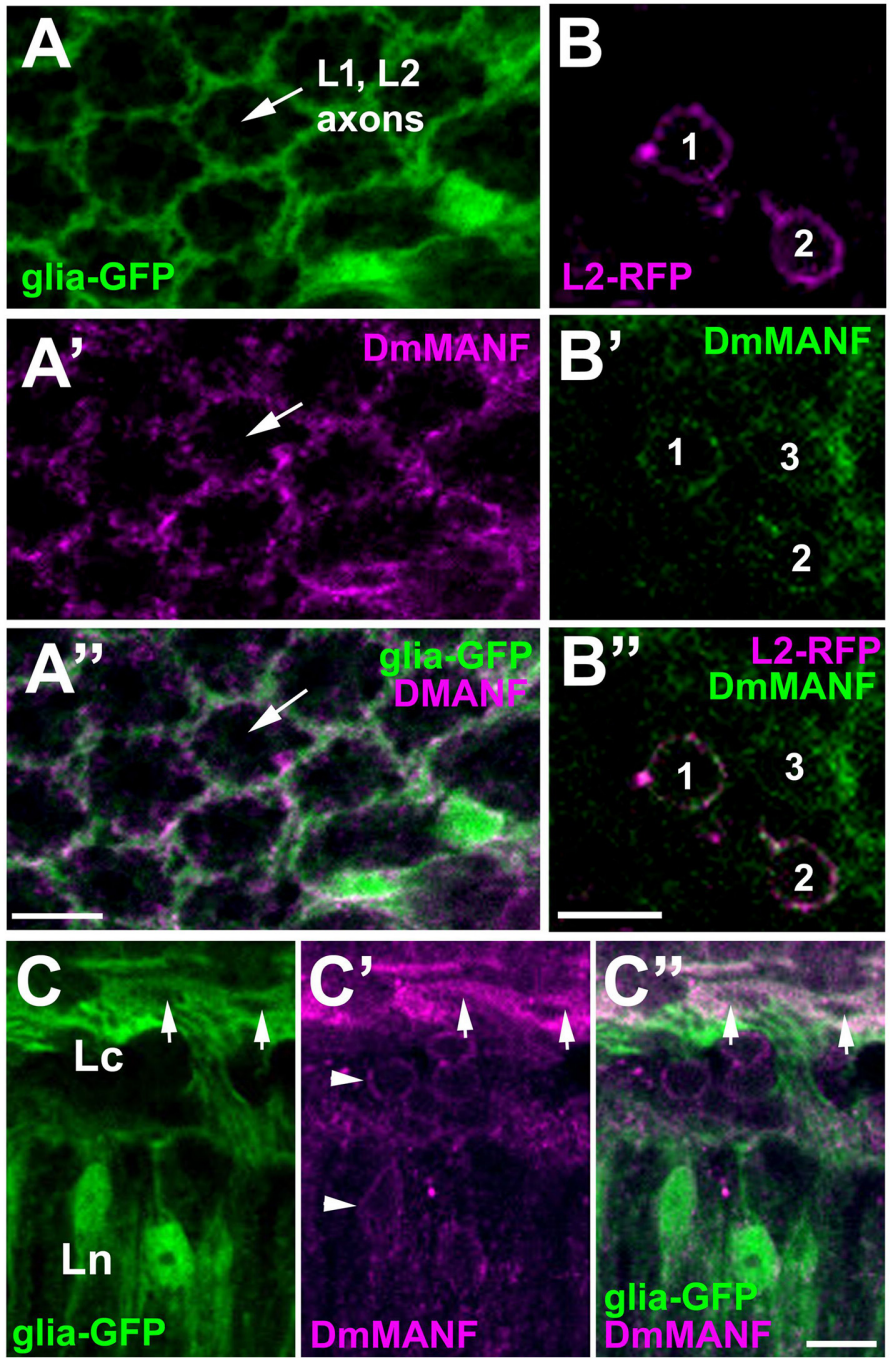
Confocal images of the lamina of *Drosophila* transgenic flies showing targeted expression of either green fluorescent protein (GFP) to glial cells [**(A,C)** groups], or red fluorescent protein (RFP—magenta) to L2 interneurons (a group of **B**), and immunolabeled with anti-DmMANF antibody. **(A–A”)** In the lamina neuropil, the pattern of DmMANF staining **(A')** resembles the pattern of GFP expression in glia **(A)**, suggesting that it is present predominantly in their processes. The center of a cartridge, where the axons of L1 and L2 large monopolar cells reside shows no staining. **(B–B”)** In the lamina cortex, DmMANF-specific immunofluorescence is observed in cell bodies (1–3) of many cells **(B')**, including L2 monopolar cells (1 and 2) **(B,B”)**. **(C–C”)** Strong staining of DmMANF in the surface glia (arrows) of the lamina cortex in comparison with the subtle perinuclear staining in other cells of the lamina cortex and neuropil (arrowheads in **C'**). Scale bars: 5 μm.

The fact that DmMANF was found to be present not only in the epithelial glial cell somata (like in neurons) but also in their long processes enveloping the synaptic cartridges (Figures 2C,D, 4A–A”), implies that it might be involved in lamina functions, which are controlled predominantly by glia. In the case of neurons, for example the L2 interneurons that reside in the center of each cartridge and receive photic and visual information from photoreceptors (Figure 1), DmMANF was present only in the perinuclear region of their somata (Figures 4B–B”). Interestingly, the particularly strong staining of DmMANF was also visible in the surface glia of the lamina cortex. The cytoplasm of their somata appeared to be loaded with DmMANF (Figures 4C–C”).

### Silencing of *DmMANF* in glial cells causes glial neurodegeneration

**Figure 5:**
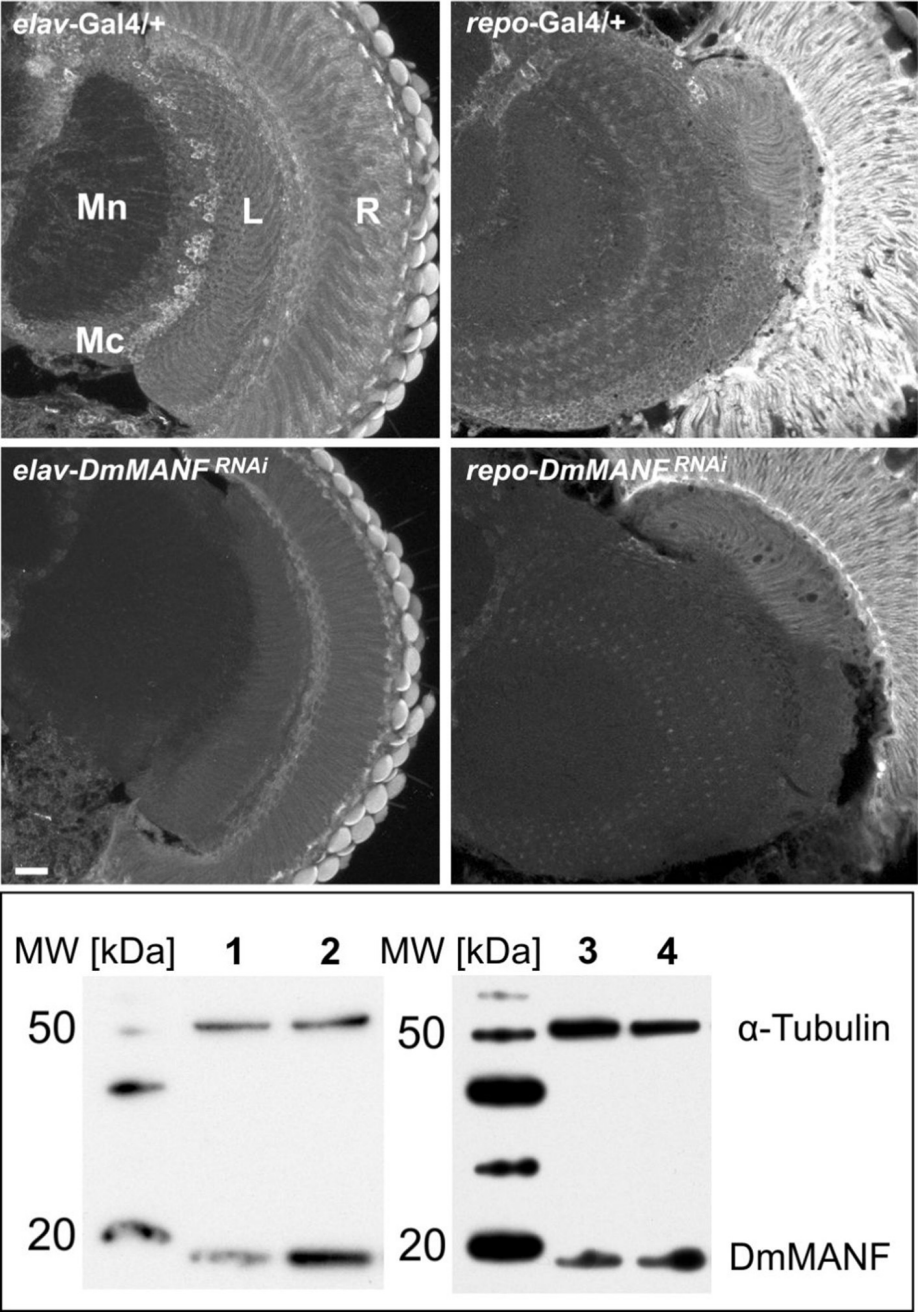
Immunohistochemical detection (upper panel) and Western blotting analysis (lower panel) of DmMANF in control (*elav-*Gal4*/*+ and *repo-*Gal4*/*+), *elav-*Gal4>UAS*-DmMANF*^*RNAi*^ and *repo-*Gal4>UAS*-DmMANF*^*RNAi*^ flies. (**Upper panel**) DmMANF-specific immunofluorescence is lower in brains with silenced expression of *DmMANF* than in control brains. Scale bar: 20 μm. R, retina; L, lamina; Mn, medulla neuropil; Mc, medulla cortex. (**Lower panel**) The level of DmMANF in whole head homogenates after *DmMANF* silencing in neurons (lane 1, *elav-*Gal4>UAS-*DmMANF*^*RNAi*^) and in glia (lane 3, *repo-*Gal4>UAS-*DmMANF*^*RNAi*^) was decreased in comparison with control lines (line 2, *elav-*Gal4/+; line 4, *repo-*Gal4/+, respectively). The anti-DmMANF Ab labels a band at 19 kDa. α-Tubulin was used as a loading control.

To determine the importance of DmMANF in neurons and glial cells, we decreased the level of DmMANF in one of these two types of cells by expressing *DmMANF*^*RNAi*^ under either a pan-neuronal (*elav*) or pan-glial (*repo*) promoter. Next, we examined the total level of DmMANF in the head and the structure of the brain. Western blot analysis confirmed the silencing of *DmMANF* in both instances, using either the neuronal driver (*elav*-Gal4>UAS*-DmMANF*^*RNAi*^ flies-80% decrease of the protein level) or the glial driver (*repo*-Gal4>UAS*-DmMANF*^*RNAi*^ flies-74% decrease of the protein level) (Figure 5). In both cases, considerable knockdown of the DmMANF protein was observed in the head lysates, but in the case of the neuronal driver, the downregulation was slightly stronger (Figure 5).

**Figure 6:**
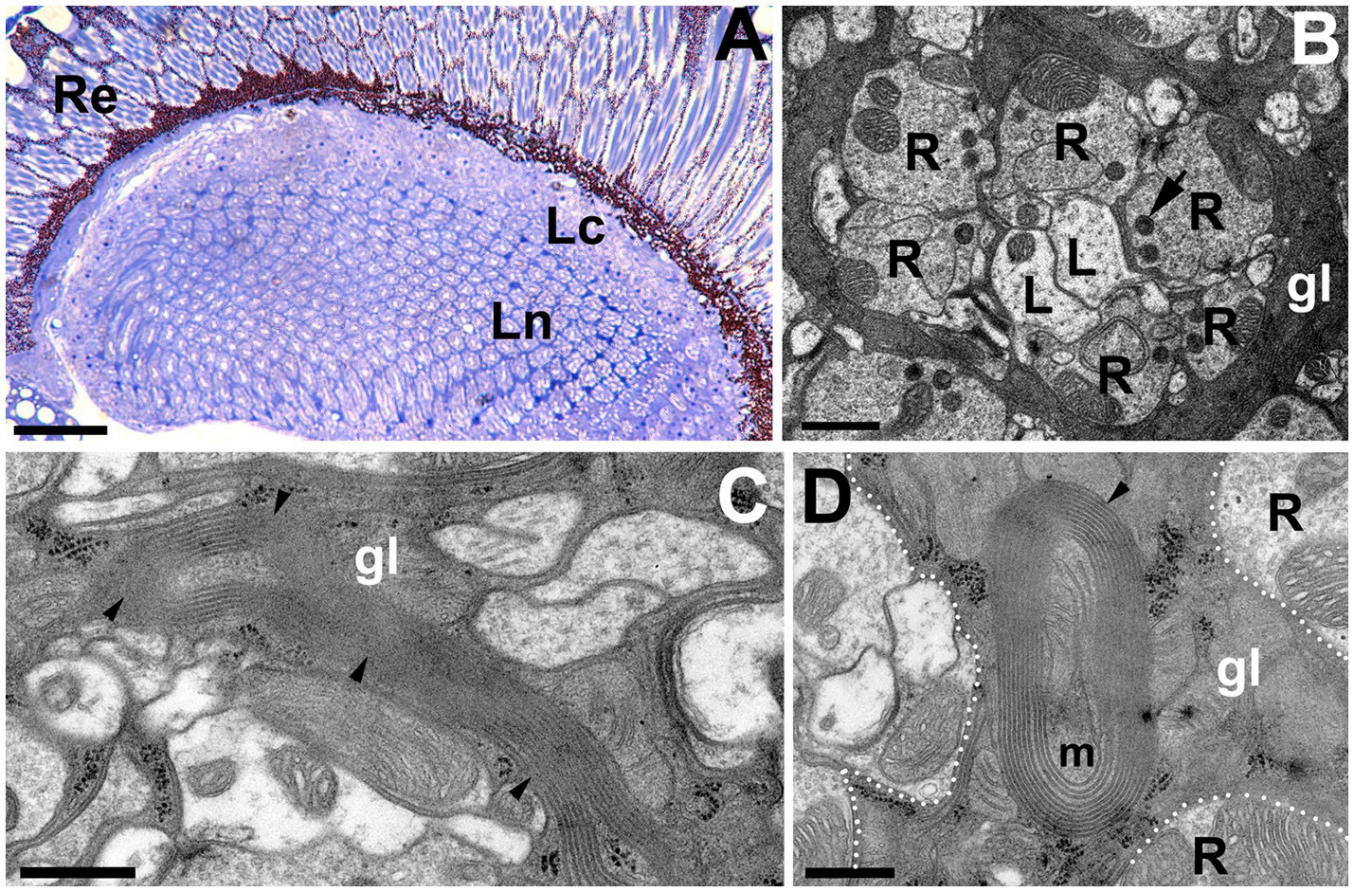
The structure of the first visual neuropil or lamina of *elav-*Gal4>UAS*-DmMANF*^*RNAi*^ flies. **(A,B)** LM **(A)** and EM **(B)** micrographs of *elav-*Gal4>UAS*-DmMANF*^*RNAi*^ lamina revealing no signs of neurodegenerations. R-photoreceptor terminals of the lamina cartridge, L-axons of L1 and L2 monopolar cells, gl-processes of the epithelial glia, which send projections into photoreceptor terminals, the so called capitate projections (arrow). Scale bar for **(A)**: 25 μm and for **(B)**: 1 μm. **(C)** Long sheets of ER in glial cells of *elav-*Gal4>UAS*-DmMANF*^*RNAi*^ tissue. Scale bar: 500 nm. **(D)** The mitochondrion (m) enveloped by ER membranes from the epithelial glia (gl). Re, retina; Lc, lamina cortex; Ln, lamina neuropil. Scale bar: 500 nm.

**Figure 7:**
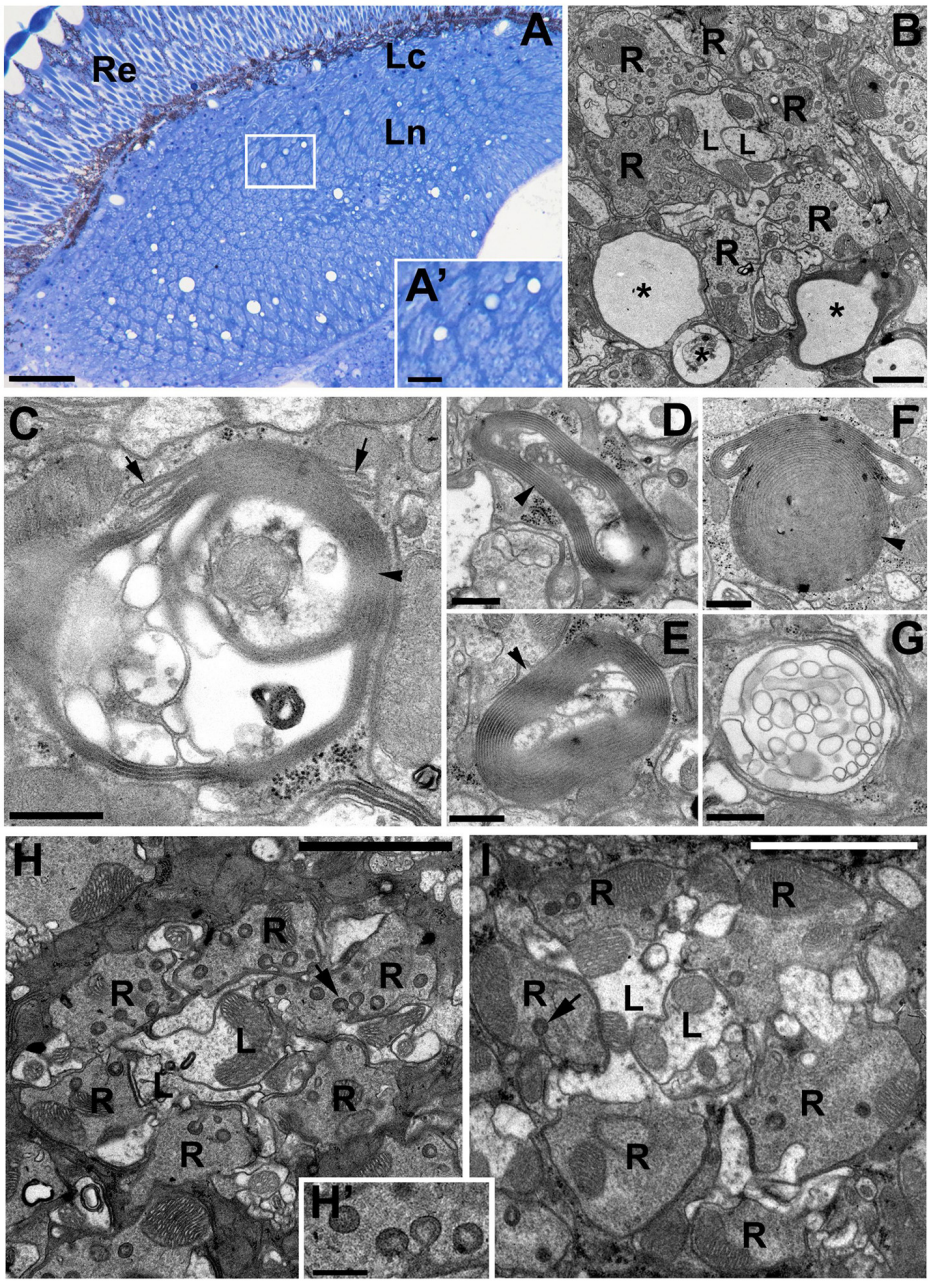
The degenerative changes in the lamina neuropil after *DmMANF* silencing in glial cells of *repo-*Gal4>UAS*-DmMANF*^*RNAi*^ transgenic flies. **(A)** The semi-thin cross-section of the lamina showing numerous holes in the neuropil region. Importantly, they are localized exclusively between the lamina cartridges—in the area occupied by epithelial glial cells (insert **A'**). Re, retina; Lc, lamina cortex; Ln, lamina neuropil. Scale bar for **(A)** 25 μm and for **(A')**: 5 μm. **(B)** EM micrograph of a single cartridge surrounded by glia with multiple holes (asterisks). R-photoreceptor terminals, L-L1 and L2 cells. Scale bar: 1 μm. **(C–G)** Glial processes contain also membrane folds (arrowheads) and/or vacuolizations, which appear to be the part of endoplasmic reticulum (**C**, arrows). Scale bar for **(C–G)**: 500 nm. **(H,I)** The EM micrographs of *repo-*Gal4*/*+ control flies **(H)** and *repo-*Gal4>UAS*-DmMANF*^*RNAi*^ **(I)** cartridges indicate that the latter **(I)** frequently bear a reduced number of glia projections (arrows). **(H')** The magnification of capitate projections from **(H)**. R-photoreceptor terminals, L-L1 and L2 cells. Scale bar for **(H,I)**: 3 μm and for (**H')**: 500 nm.

Light microscopy (LM) and TEM examinations of the adult brains with silenced expression of *DmMANF* in neurons (*elav-*Gal4>UAS-*DmMANF*^*RNAi*^) or glia (*repo-*Gal4>UAS-*DmMANF*^*RNAi*^) revealed that while the structure of the former brains showed no apparent changes compared with that of the brains of control flies (Figures 6A,B), the structure of the latter brains displayed signs of degeneration (Figures 7A–G). Therefore, a glia autonomous function of DmMANF with respect to degeneration was demonstrated. Clear signs of degeneration were observed as holes of different size. Even though the silencing of *DmMANF* was slightly less efficient in glia than in neurons, it had a stronger effect. Interestingly, the observed holes were located between cartridges, in the region occupied by processes and cell bodies of the epithelial glial cells (Figures 7A,B). TEM studies showed that these structures were surrounded by pronounced membranous folds belonging to ER (Figures 7C–G). Moreover, compared with control *repo-*Gal4*/*+ flies (Figure 7I), *repo-*Gal4>UAS-*DmMANF*^*RNAi*^ flies showed a strong decrease (30%) in the number of capitate projections (Figures 7H,H').

In contrast to pan-neuronal silencing of the expression of *DmMANF* (Figures 6A,B), its silencing in photoreceptors only (*GMR-*Gal4>UAS-*DmMANF*^*RNAi*^ and *RH1-*Gal4>UAS-*DmMANF*^*RNAi*^) or in L2 monopolar cells (*21D-*Gal4>UAS-*DmMANF*^*RNAi*^), which normally express DmMANF, did not lead to neurodegeneration (data not shown). The pan-neuronal silencing of *DmMANF* caused only sporadic occurrence of long membrane sheets of ER (Figure 6C) or folds surrounding e.g., mitochondria (Figure 6D). The latter observation suggests signs of autophagy (mitophagy) (reviewed i.e., in Knuppertz and Osiewacz, 2016). A decrease in the capitate projection number was also detected (data not shown).

**Figure 8:**
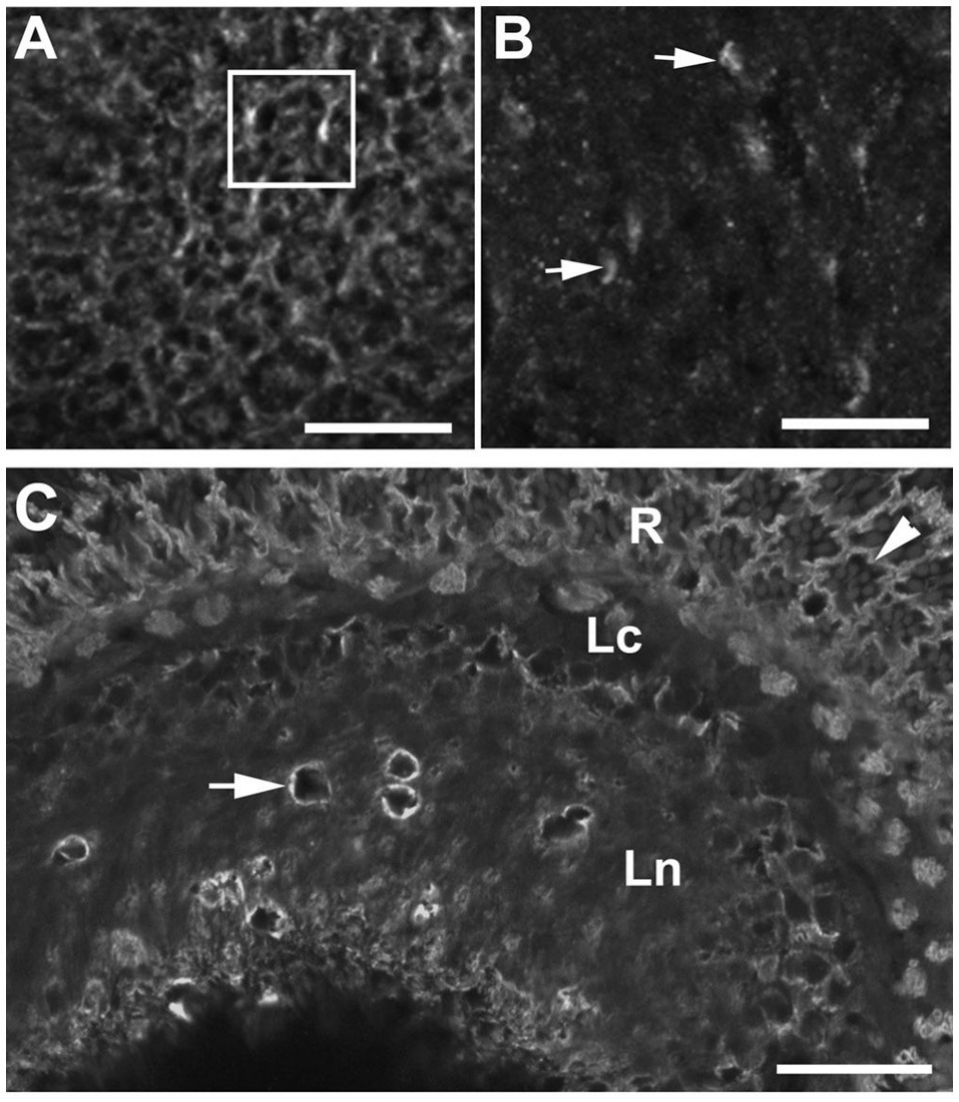
The immunolabeling pattern of α-subunit of Na^+^/K^+^-ATPase α-subunit in the lamina neuropil of *repo-*Gal4*/*+ control flies **(A)** and *repo-*Gal4>UAS*-DmMANF*^*RNAi*^ flies **(B)**. In the lamina of control flies **(A)**, membranes containing the α-subunit of Na^+^/K^+^-ATPase are well visible in the localization of epithelial glia and other cells, and therefore cartridges (rectangle-a single cartridge) can easily be discerned. In the lamina of experimental flies with reduced expression of DmMANF in glia **(B)**, on the other hand, cartridges can no longer be distinguished. Numerous spots of stronger but diffuse fluorescence (arrows) can be observed in the place of punctures. Scale bar: 10 μm. **(C)** Although the distribution of Na^+^/K^+^-ATPase α-subunit is disturbed in the lamina neuropil (Ln), its presence at the basolateral membrane of photoreceptors (arrowhead) of the retina (R) and the expression in the lamina cortex (Lc) are not affected. Degenerative changes are surrounded by membranous aggregates strongly labeled against α-subunit of sodium-potassium pump (arrow). Scale bar: 20 μm.

Because the epithelial glial cells exhibited massive membranous folds, we also examined how silencing of *DmMANF* in glia influences the membranous expression of the α-subunit of Na^+^/K^+^-ATPase in cells of the lamina (Figure 8). Its expression is known to display circadian changes (Górska-Andrzejak et al., 2009). Interestingly, we found that the distribution of the α-subunit of Na^+^/K^+^-ATPase in the lamina neuropil was highly disorganized, and the lamina cartridges could no longer be distinguished (Figures 8B,C). The silencing of *DmMANF* in glia disrupted the distribution of the α-subunit of the sodium-potassium pump in the epithelial glia and other cells and consequently the lamina's functions.

### The reduced DmMANF level in glia influences life expectancy and locomotor behavior of flies

**Figure 9:**
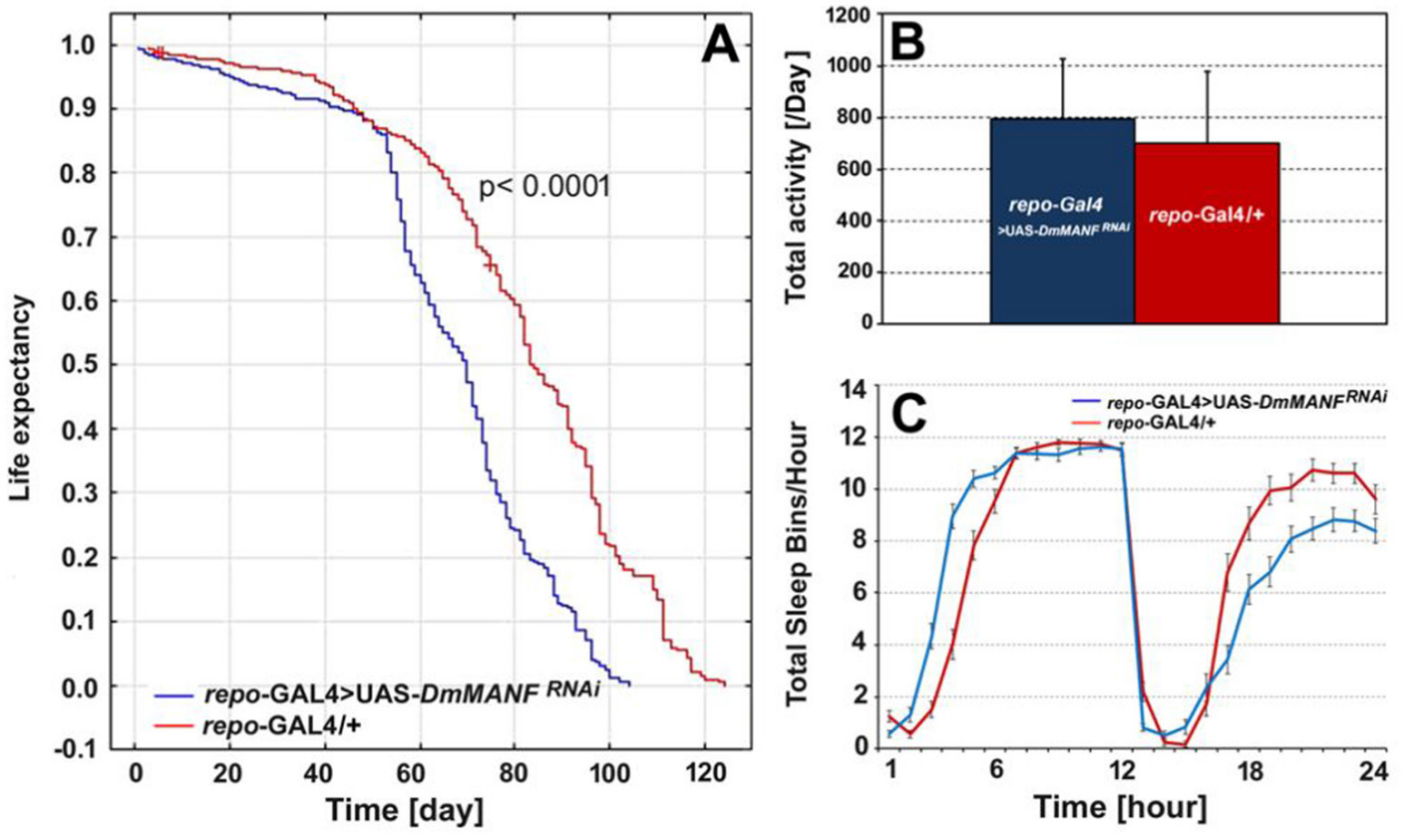
The comparison of life expectancy **(A)**, the total level of activity **(B)** and total sleep bins during the day and night **(C)** of *repo*-Gal4>UAS-*DmMANF*^*RNAi*^ and the control, *repo*-Gal4/+ flies. The total level of activity [**(B)**, mean + SD] and the sleep analysis [**(C)**, mean ± SE] was conducted in light/dark conditions LD 12:12 and counted for the second day of activity recording.

Analyses of life expectancy (adult lifespan) and locomotor activity parameters such as the level of activity and the sleep/activity ratio revealed changes after silencing of *DmMANF* in glia (Figure 9). Compared with the lifespan of control males (*repo-*Gal4*/*+; *N* = 365), the lifespan of males with silenced expression of *DmMANF* in glial cells (*repo*-Gal4>UAS-*DmMANF*^*RNAi*^; *N* = 328) was reduced by 16% (log-rank test, *p* < 0.0001). The median life expectancy of *repo*-Gal4>UAS-*DmMANF*^*RNAi*^ flies was 70 days, while *repo*-Gal4*/*+ control flies lived 83 days (Figure 9A). However, the overexpression of *DmMANF* in glial cells of *repo*-Gal4>UAS-*DmMANF* flies did not extend the lifespan of these flies (data not shown).

The mean activity level of *repo-*Gal4>UAS*-DmMANF*^*RNAi*^ flies did not differ significantly compared with that of control flies (Mann-Whitney *U*-test, *p* = 0.07) (Figure 9B). However, in most *repo-*Gal4>UAS*-DmMANF*^*RNAi*^ flies we observed a 9% decrease of activity in the light phase of the cycle (Mann-Whitney *U*-test, *p* = 0.003) and a 20% increase of activity in the dark phase (Mann-Whitney *U*-test, *p* = 0.001). Flies with silenced expression of *DmMANF* in glia had fewer sleep bins per hour during the dark phase of the LD 12:12 cycle and slightly more at the very beginning of the day (the light phase of the cycle) (Figure 9C).

**Figure 10:**
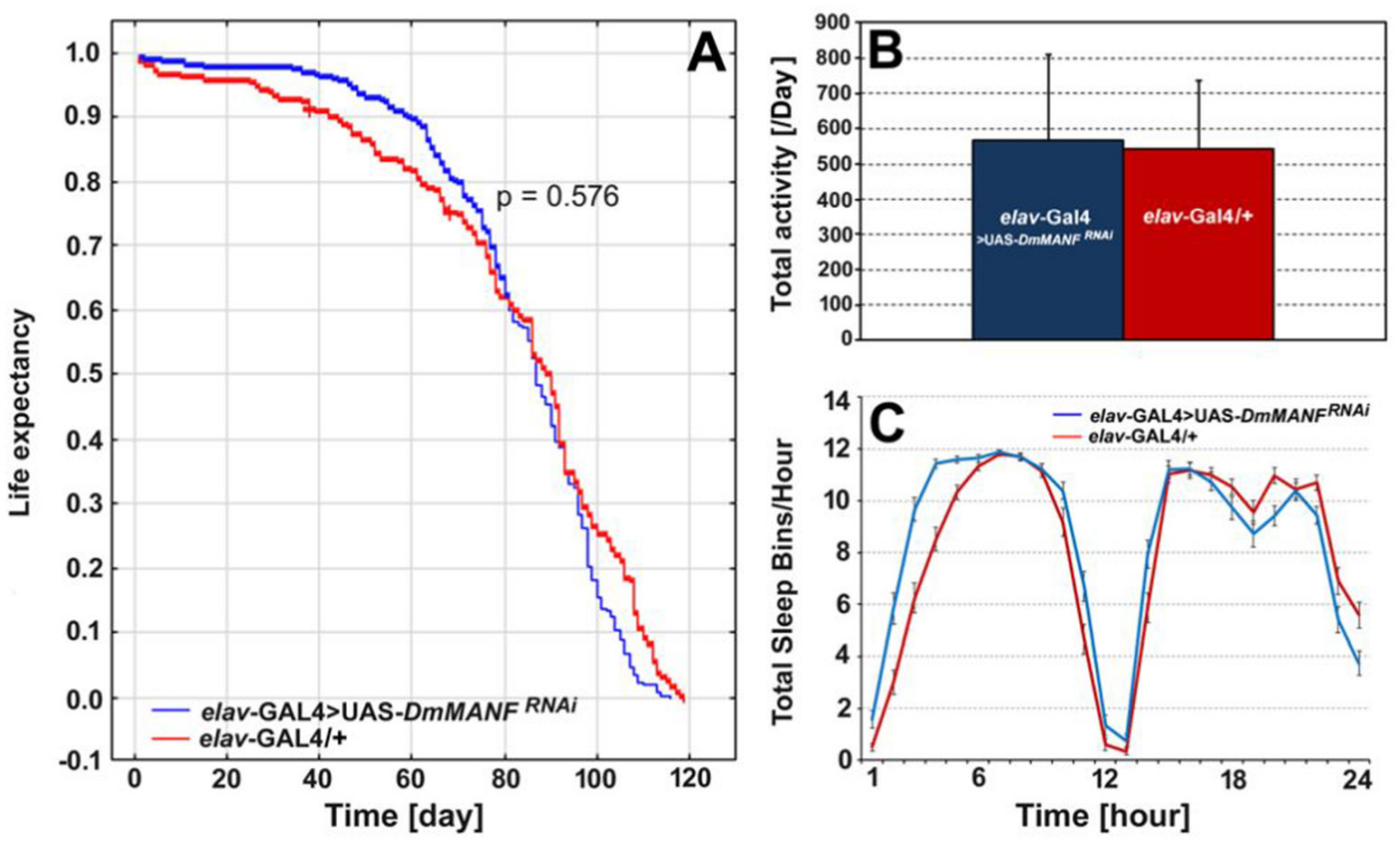
The comparison of life expectancy **(A)**, the total level of activity **(B)** and total sleep bins during the day and night **(C)** of *elav*-Gal4>UAS-*DmMANF*^*RNAi*^ and control, *elav*-Gal4/+ flies. The total level of activity [**(B)**, mean + SD] and the sleep analysis [**(C)**, mean ± SE] was conducted in light/dark conditions LD 12:12 and counted for the second day of activity recording.

The lifespan of males with silenced expression of *DmMANF* in neurons (*elav*-Gal4>UAS-*DmMANF*^*RNAi*^ flies; *N* = 320), on the other hand, was not significantly different from the lifespan of control flies (*elav-*Gal4/+; *N* = 203) (Figure 10A). Furthermore, overexpression of *DmMANF* in neurons (*elav*-Gal4>UAS-*DmMANF*) had no significant influence on the flies' lifespan (data not shown). There were also no significant differences in the total level of locomotor activity after decreasing the level of *DmMANF* expression in neurons (Mann-Whitney *U*-test, *p* = 0.8; Figure 10B). In agreement with the flies with silenced expression of DmMANF in glia, *elav-*Gal4>UAS*-DmMANF*^*RNAi*^ males exhibited decreased activity in the light phase of the cycle (Mann-Whitney *U*-test, *p* = 0.0004) (Figure 10C).

## Discussion

DmMANF immunoreactivity has been detected in both neurons and glial cells of the *Drosophila* adult brain (Stratoulias and Heino, 2015a). Our results show that in neurons the decrease of DmMANF level does not lead to visible defects in the brain structure (Figures 6A,B), while in glia it drives degeneration. This degeneration manifests as holes mainly in the first visual neuropil (lamina) (Figure 7), where DmMANF is strongly expressed (Figure 2A). In addition, silencing of *DmMANF* expression in glia significantly disrupts the membrane distribution of the α-subunit of the Na^+^/K^+^-ATPase in the lamina. The abundance of this protein, predominantly in the epithelial glial cells, has been shown to reveal circadian changes correlated with other circadian rhythms in the lamina (Pyza and Meinertzhagen, 1999; Pyza and Górska-Andrzejak, 2004; Górska-Andrzejak et al., 2009; Weber et al., 2009; Damulewicz et al., 2013).

Interestingly, the degeneration found was in the epithelial glial cells, the neuropil glia that envelop the synaptic units (cartridges) of the lamina throughout its depth. The signs of degeneration were observed either as small holes encircled by membranous aggregates or piles of ER membranes (Figures 7B–G). This finding suggests that reduction of DmMANF causes ER stress in the epithelial glial cells. A cytoprotective function of MANF in ER stress has been reported in many studies (Apostolou et al., 2008; Tadimalla et al., 2008; Lindahl et al., 2014), and it has been suggested that MANF is able to bind to lipids or membranes in the ER stress response (Parkash et al., 2009). Moreover, in *DmMANF* mutants, the expression of several genes involved in UPR is significantly changed (Palgi et al., 2012).

Taking into consideration that: (i) DmMANF can bind to membranes (Parkash et al., 2009), (ii) it localizes in the ER (Palgi et al., 2012) (Figures 2E,E'); and above all, (iii) the cell bodies and processes of the epithelial glial cells contain a very elaborate network of ER (Figure 3), it is likely that DmMANF has a critical role in the activity of epithelial glia. Therefore, reduced DmMANF levels in glia might first and foremost cause epithelial glial cell degeneration. The ultrastructure of the epithelial glial cells strongly implies that a decrease in DmMANF may activate autophagy because vesicles, small neuronal processes or mitochondria were detected in many cartridges, inside the piles of glial membranes (Figures 7C–G). This result is in agreement with other studies showing that autophagy is essential for the survival of astrocytes (Korenić et al., 2015) and dysfunction of autophagy causes neurodegeneration (Liang and Le, 2015). Interestingly, silencing of *DmMANF* expression (together with *Dicer-2* expression) or induction of either immunity or autophagy specifically in *Drosophila* glial cells results in the appearance of the so-called MANF-immunoreactive Cells (MiCs), which resemble vertebrate microglia–neither microglia nor microglia-like cells have been found in *Drosophila* before (Stratoulias and Heino, 2015b). In contrast, MiCs were not found after *DmMANF* silencing using the pan-neuronal *elav*-Gal4 driver (Stratoulias and Heino, 2015b). In accordance, in this study we did not observe neurodegeneration in the lamina following *DmMANF* silencing in neurons (Figure 6A).

Apart from degeneration, we observed a lower number of capitate projections in the cartridges of flies with silenced expression of *DmMANF* in glia (Figures 7H,I). Capitate projections of the epithelial glial cells, which invaginate into the photoreceptor terminals (Trujillo-Cenóz, 1965; Stark and Carlson, 1986; Prokop and Meinertzhagen, 2006), are suggested to be involved in the recycling of histamine, a neurotransmitter in tetrad synapses of photoreceptors (Hardie, 1987; Meinertzhagen and O'Neil, 1991; Fabian-Fine et al., 2003). It is known that *de novo* synthesis of histamine is a relatively slow process. Histamine released in tetrad synapses is thus taken up by the epithelial glial cells and inactivated through β-alanine conjugation by the enzyme Ebony (*N*-β-alanyldopamine synthase). Its inactive metabolite, carcinine, is then transported back into photoreceptors (Morgan et al., 1999; Borycz et al., 2002; Richardt et al., 2003; Xu et al., 2015). The decreased number of epithelial glia capitate projections (Figures 7H,I), which facilitate this transport (Rahman et al., 2012), must therefore impair transmission of photic and visual information between the photoreceptors (R1-R6) and the first-order interneurons in the lamina. The inability of the epithelial glial cells to form the usual number of capitate projections without the appropriate amount of DmMANF confirms that in such conditions they no longer maintain their functions at adequate levels. Surprisingly, however, silencing of *DmMANF* in neurons (after *elav*-driven silencing of *DmMANF*) had a similar effect—it decreased the number of capitate projections. This indicates that glial cells are capable of diagnosing the lowered level of DmMANF in neurons and that the number of capitate projections depends not only on the condition of glia but also that of their neuronal partners. The neurons of the cartridge, particularly the photoreceptors into which glia invaginate, may not function at their best even though their ultrastructure does not reveal strong signs of degeneration.

Although the silencing of *DmMANF* in neurons does not lead to strong phenotypes in the brain areas studied here, TEM analysis demonstrated not only a decrease in the number of capitate projections but also the occurrence of sporadic membrane-rich structures (sometimes with mitochondria inside—mitophagy) in the surrounding epithelial glial cells (Figures 6C,D). These changes from neuronal *DmMANF* downregulation were mild compared with those from glial *DmMANF* downregulation. The induction of mitophagy in the case of epithelial glial cells that have their cytoplasm filled with numerous mitochondria (Figure 3; which implies a high level of metabolism) may indicate changes in the level of glia metabolism in response to changes in neuronal physiology (Patergnani and Pinton, 2015). Despite the fact that strong expression of DmMANF was observed in the L2 interneuron and photoreceptor cell bodies, neurodegenerative changes were not observed in flies with silenced expression of *DmMANF* in either L2 (using *21D*-Gal4 driver) or photoreceptors (using *GMR*-Gal4 or *RH1*-Gal4 lines). DmMANF localization in their somata, but not in processes similar to in the case of epithelial glial cells, suggests however that in neurons it may be important for neurotransmitter and protein synthesis.

It might be puzzling why *DmMANF* silencing in neurons did not cause neurodegeneration (Figure 6) while its decrease in glia did (Figure 7). Nevertheless, it has been shown that dysfunction of glia often occurs to be the first step in neurodegeneration of the nervous system and can lead to pathological processes in the brain (reviewed in Heneka et al., 2010). Similar neurodegenerative phenotypes have been observed in glial cells of *swiss cheese* (*sws*) (Kretzschmar et al., 1997; Dutta et al., 2016) and *drop-dead* (Buchanan and Benzer, 1993) mutants. These flies also exhibited vacuolization and multilayered glial sheaths, which highly affected the nervous system and finally caused the death of the flies. RNAi-mediated knockdown of *sws* gene expression under control of the pan-glial promoter *repo* triggered similar degenerative changes although in all glial cells (Dutta et al., 2016). Glial degeneration is also a characteristic of the fruit fly model of human Ataxia-Telangiectasia (ATM) disease (Petersen et al., 2013).

It also seems that the glial cells that are particularly sensitive to presence of DmMANF belong to the neuropil glia. We have not observed such degenerative changes in other types of glia in the brain even though DmMANF is present at high levels in subperineurial (SPG) and perineurial (PG) glia (Stratoulias and Heino, 2015a). These two types of glial cells form the two layers surrounding the fly brain. They shield neurons from haemolymph and form, at least partly, the blood-brain barrier (BBB). In turn, SPG cells form septate junctions and prevent paracellular diffusion (Stork et al., 2008; DeSalvo et al., 2014; Limmer et al., 2014). We also observed high levels of DmMANF in the surface glia, which build a barrier between the retina and the optic lobe of the brain. It is possible that the BBB in flies with decreased levels of DmMANF might not function properly in detoxifying the brain, which consequently might lead to the shortened lifespan and disturb the day/night sleep pattern (Benveniste et al., 2017).

Apart from the analyses of phenotype at the cellular level, we also examined the lifespan and total activity/sleep in flies with decreased levels of DmMANF in glia or in neurons. Similar to *sws* (Kretzschmar et al., 1997), *drop-dead* (Buchanan and Benzer, 1993) and the *Drosophila* model of ATM disease (Petersen et al., 2012), males with silenced expression of *DmMANF* in glia showed reduced lifespan. They also slept less in the dark phase (night) and more in the light phase (day) of the LD 12:12 cycle. Such differences in sleep duration may result from the disruption of neurotransmitter turnover in the brain. Microarray studies of *DmMANF* mutants have shown downregulation of genes encoding membrane transporters and genes involved in amine catabolic processes (Palgi et al., 2012). Thus, the low level of DmMANF in glia may alter the level of dopamine and other neurotransmitters that regulate *Drosophila* behavior (Buchanan and Benzer, 1993). In turn, the life expectancy of flies with *DmMANF* downregulation in neurons was not significantly different from the life expectancy of control flies. However, they showed decreased locomotor activity in the light phase of LD 12:12.

Embryos lacking both maternal and zygotic *DmMANF* die as late embryos and show drastic phenotypes in the nervous system (Palgi et al., 2009), indicating the important role of DmMANF in the nervous system. Our results strongly suggest a pivotal role of DmMANF in homeostasis of glia in general, and neuropil glia in particular. They also reveal that the *Drosophila* lamina may be a good model for studying DmMANF function due to the presence of the epithelial glial cells, which show particular sensitivity to DmMANF. It also appears to be a good model for studying the role of DmMANF in neuron–glia interactions in the synaptic neuropil, since glial DmMANF influences neuronal homeostasis and neuropil functioning. The wide range of DmMANF modes of function, including its therapeutic potential in neurodegenerative diseases (Voutilainen et al., 2015), can be studied using this model.

## Ethics statement

We used *Drosophila melanogaster* for our study and studies on insects do not have to be approved by a committee approving research on animals.

## Author contributions

LW, EK, WK, JG, OW, and EC carried out experiments, analyzed data, prepared figures. VS and TH provided Drosophila lines and antibodies. LW, WK, JG, EP wrote the manuscript. All authors checked and discussed the manuscript.

### Conflict of interest statement

The authors declare that the research was conducted in the absence of any commercial or financial relationships that could be construed as a potential conflict of interest.
